# Maternal and Perinatal Outcomes in Pregnant Women with Cystic Fibrosis

**DOI:** 10.1055/s-0039-1678613

**Published:** 2019-03-07

**Authors:** Gilmar de Souza Osmundo Junior, Rodrigo Abensur Athanazio, Samia Zahi Rached, Rossana Pulcineli Vieira Francisco

**Affiliations:** 1 Faculty of Medicine, Division of Obstetrics, Department of Obstetrics and Gynecology, Hospital das Clínicas da Universidade de São Paulo, SP, Brazil; 2 Faculty of Medicine, Division of Pneumology, Heart Institute, Hospital das Clínicas da Universidade de São Paulo, SP, Brazil

**Keywords:** cystic fibrosis, pregnancy complications, respiratory infections, fibrose cística, complicações na gravidez, infecções respiratórias

## Abstract

**Objectives** To assess the perinatal and maternal outcomes of pregnant women with cystic fibrosis (CF) and severe lung impairment.

**Methods** This was a series of cases aiming to review the maternal and fetal outcomes in cases of singleton pregnant women with a diagnosis of CF. We have included all of the cases of singleton pregnancy in patients with CF who were followed-up at the obstetrics department of the Medical School of the Universidade de São Paulo, between 2003 and 2016. The exclusion criteria were the unattainability of the medical records of the patient, and delivery at other institutions. A forced expiratory volume in 1 second < 50% was considered as severe lung impairment. We have also analyzed data regarding maternal hospitalization and respiratory exacerbations (REs).

**Results** Pregnant women with CF and severe lung impairment did not present an association with spontaneous prematurity, fetal growth restriction or fetal demise. All of the cases involved multiple RE episodes requiring antibiotic therapy. The median (range) of events per patient was of 4 (2–4) events.

**Conclusion** Cystic fibrosis patients with severe lung impairment may achieve successful term pregnancies. However, pregnancies of women with CF are frequently complicated by REs, and this population may require hospital admission during the course of the pregnancy. Cystic fibrosis patients should be followed by a specialized team with experience in treating respiratory diseases.

## Abstract (pt)

**Resumo:**

**Objetivo** Avaliar os desfechos maternos e perinatais de gestações em mulheres portadoras de fibrose cística (FC) e disfunção pulmonar grave.

**Métodos** Série de casos visando a avaliação dos desfechos maternos e perinatais em gestações únicas de mulheres com diagnóstico de FC. Foram incluídos todos os casos de gestações únicas de pacientes com FC que tiveram acompanhamento no departamento de obstetrícia da Faculdade de Medicina da Universidade de São Paulo, no período de 2003 a 2016. Os critérios de exclusão foram não disponibilidade do prontuário da paciente e parto em outro serviço. Disfunção pulmonar grave foi definida como presença de volume expiratório forçado em 1 segundo < 50%. Foram analisados também os dados referentes a exacerbações respiratórias e internações maternas.

**Resultados** Gestação em mulheres portadoras de FC com disfunção pulmonar grave não se associaram com prematuridade espontânea, restrição de crescimento fetal ou óbito fetal. Todos os casos apresentaram múltiplos episódios de exacerbações respiratórias necessitando de antibioticoterapia. A mediana de eventos por pacientes (intervalo) foi de 4 (2–4) eventos.

**Conclusão** Mulheres portadoras de FC com disfunção pulmonar grave podem evoluir com gestações de termo bem sucedidas. Entretanto, gestações nestas pacientes são frequentemente complicadas por exacerbações respiratórias, necessitando de internação. Gestantes portadoras de FC devem ser acompanhadas por uma equipe especializada com experiência no manejo de doenças respiratórias.

## Introduction

Cystic fibrosis (CF) is an inherited autosomal recessive disease with a worldwide incidence of 1 in between 2,000 and 5,000 live births, and an incidence of ∼ 1 in 7,000 live births in the Brazilian population.^1^ ^2^ Genetic mutations in chromosome 7 lead to impaired transepithelial chloride transport and thickened secretions in the respiratory and digestive tracts.^3^ ^4^ Cystic fibrosis patients may present with recurrent respiratory infections, bronchiectasis, progressive deterioration of the lung function, exocrine pancreatic insufficiency, and CF-related diabetes.^4^

The life expectancy of CF patients has increased markedly in the recent decades.^5^ Currently, the predicted survival of an American CF patient is of 41.7 years, and it has been estimated that infants born in the 2000s will survive beyond the age of 50 years old.^5^ ^6^ As the life expectancy and the quality of life improve, the pregnancy rate has also been increasing. The US Cystic Fibrosis Foundation reported 235 pregnant women with CF in 2015.^5^ Fair et al^7^ assessed attitudes toward fertility among CF adult patients and found that 72% of the women with CF considered having children to be an important issue.

The first described pregnancy in a woman with CF resulted in a preterm newborn and in maternal death 6 weeks after the delivery.^8^ Subsequent studies showed poor maternal outcomes and high prematurity and miscarriage rates.^9^ ^10^ In the 1980s, a maternal death rate as high as 15% within the first 2 years of delivery was reported in this population.^11^

As the therapeutic options for CF have advanced, recent studies have demonstrated better perinatal and maternal outcomes. Goss et al,^12^ in a matched parallel-cohort study including 680 pregnant women with CF, demonstrated that neither the predicted forced expiratory volume in 1 second (FEV1) decline rate nor the survival rate were negatively influenced by the pregnancy.^12^ Girault et al^13^ observed no differences in the age of delivery, birthweight, Apgar score, cord pH or cesarean section rates between CF patients and healthy women. Nevertheless, the available data are still inconsistent regarding low birthweight and prematurity in the offspring of women with CF with severe lung function impairment.^14^ ^15^

Considering the inconclusive available information regarding pregnant women with CF and severe lung disease, as well as the lack of studies on the Brazilian population and on the profile of airway microbial colonization in Brazilian CF patients, the present study aims to evaluate the perinatal and maternal outcomes of pregnant women with CF and severe lung impairment who were followed-up at a Brazilian adult CF referral center.

## Methods

We have analyzed a series of cases comprising all of the cases of singleton pregnant women with a diagnosis of CF at the obstetrics department of the Medical School of the Universidade de São Paulo, between 2003 and 2016. The maternal and fetal outcomes in this group of patients were reviewed. The hospital is a referral center for pathologic pregnancies. A team consisting of obstetricians with experience with pregnant women with lung diseases, CF respiratory physicians, anesthesiologists, dietitians, and chest physiotherapists provided care during the pregnancy. The charts were systematically reviewed, and we have collected data regarding maternal age, weight gain, gestational age at delivery, birthweight, Apgar scores, route of delivery, maternal admissions, intensive care admissions, respiratory exacerbations (REs), requirement for antibiotic therapy, and profile of airway microbial colonization. The FEV1, the forced vital capacity (FVC), and the FEV1/FVC ratio were the lung function measures considered in the analysis. Cases with an FEV1 < 50% were regarded as severe lung disease. Quantitative data were analyzed by appropriate descriptive statistics (*n,* percentage, range, mean, or confidence interval [CI]), using PASW Statistics for Windows, Version 18.0 (SPSS Inc, Chicago, IL, USA). The study protocol was registered and approved by the Institutional Ethics Review Board (CAPPesq 2.315.811) and the need for the informed consent from the patients was waived due to the retrospective nature of the study.

## Results

Seven pregnancies were identified, corresponding to five different women. None of the patients had discussed with the CF team their motherhood planning before pregnancy. At the first appointment, the patients had a median age of 24 (18–31) years old, and a median body mass index (BMI) of 19.5 (17–22) kg/m^2^. The median weight gain was of 4.1 (- 1.4–8) kg. We have not observed any cases of miscarriage, stillbirth, preeclampsia, gestational diabetes or neonatal death. The demographic results are shown in Table 1.

**Table 1:** Demographics of the study population

Variables	
Maternal age (years old), median (range)	24 (18–31)
BMI (kg/m^2^), median (range)	19.5 (17–22)
Weight gain (g), median (range)	4.1 (-1.4–8)
GA at delivery (weeks), median (range)	38 (36.8–38.8)
Mode of delivery	
Normal vaginal, n (%)	1 (14.2)
Forceps, n (%)	3 (42.9)
Cesarian, n (%)	3 (42.9)
Birthweight (g), median (range)	2,944 (2,320–3,340)
FEV1 (%), median (range)	38 (35–87)
FVC (%), median (range)	58 (37–75)
Hospital admission (days), median (range)	20.5 (2–36)
RE episodes	
Global – number of events, median (range)	2 (0–4)
FEV1 < 50%, number of events, median (range)	4 (2–4)
Medications	
Dornase alfa, n (%)	3 (42.9)
Azithromycin, n (%)	4 (57.1)
Tobramycin (inhalation), n (%)	1 (14.2)
Budesonide/Formoterol (inhalation), n (%)	4 (57.1)

Abbreviations: BMI, body mass index; FEV1, predicted forced expiratory volume in 1 second; FVC, forced vital capacity; g, grams; GA, gestational age; IQR, Interquartile Range; RE, respiratory exacerbation.

The median gestational age at delivery was of 38 (36.8–38.8) weeks. The only preterm delivery was a cesarean section (CS) of a 36 + 6-week patient who presented with deteriorating respiratory symptoms. Four women delivered vaginally (57.1%), but operative vaginal delivery was necessary in 3 of them (3/4 cases; 75%). One emergency CS occurred due to a failed forceps delivery (Table 2). Labor analgesia consisted of a combined spinal epidural block in all of the cases. We have not observed a worsening of the maternal clinical condition during labor or after the delivery.

**Table 2:** Lung function and maternal and obstetric outcomes of the pregnancies

Patient	Age (years old)	FEV1 (%)	Mode of delivery	Indication of the mode of delivery a	GA at delivery (weeks)	Birthweight (g)	RE (*n*)
1							
Pregnancy #1	27	25	Cesarean	SGA + maternal clinical deterioration	37	2,320	4
Pregnancy #2	31	33	Cesarean	Maternal clinical deterioration	36 + 6	2,680	2
2	24	50	Normal	−	38	3,160	0
3	29	38	Forceps	Maternal exhaustion	37 + 6	2,944	4
4							
Pregnancy #1	21	b	Forceps	Rotational instrumental delivery	38 + 2	3,160	2
Pregnancy #2	24	b	Forceps	Maternal exhaustion	38 + 5	2,930	0
5	18	87	Cesarean	Failed forceps	38 + 6	3,340	4

Abbreviations: FEV1, predicted forced expiratory volume in 1 second; g, grams; GA, gestational age; RE, respiratory exacerbation; SGA, small for gestational age fetus.

a Indication of cesarean or forceps delivery.

b Patient underwent unilateral pulmonary resection.

Although one case of a small for gestational age fetus occurred, this diagnosis was not confirmed after the delivery. All of the newborns were adequate for the gestational age and had a median birthweight of 2,944 (2,320–3340) g. We have not observed any cases of an Apgar score < 7, of cord pH < 7.2, or of neonatal intensive care admission.

One patient had exocrine pancreatic insufficiency, but no cases of CF-related diabetes or liver disease occurred. Regarding airway microbial colonization, we observed a prevalence of 85.7% (*n* = 6) for *Pseudomonas aeruginosa*, and a prevalence of 28.6% (*n* = 2) for *Burkholderia cepacia*. Five cases (71.4%) presented with RE. Four cases (57.1%) required hospital admission, with a median stay of 20.5 (2–36) days. The reasons for admission were RE, hemoptysis, and worsening of dyspnea (Table 3). The patients colonized by *B. cepacia* had the longest hospitalization stays (36 and 33 days).

**Table 3:** In-hospital treatment results of pregnant women with cystic fibrosis

Case	FEV1 (%)	GA (weeks)	Indication	Microbial Colonization	Stay duration (days)	Treatment a
*1*	33			*Staphylococcus aureus, Pseudomonas aeruginosa, Haemophilus influenzae*		
*Admission #1*		32	RE		6	- Oxacillin; - Ceftazidime; - RT
*Admission #2*		36 + 4	Worsening dyspnea		5	- RT; - Glucocorticoids; - Cesarean section at 36 + 6 weeks
*2*	3			*Burkholderia cepacia, Serratia marcescens Staphylococcus aureus, MRSA, Pseudomonas aeruginosa*		
*Admission #1*		18	RE		16	- Oxacillin; - Meropenem; - RT
*Admission #2*		26	Respiratory sepsis		10	- ICU; - Oxacillin; - Ceftazidime; - RT
*Admission #3*		32	RE		7	- Oxacillin; - Meropenem; - Teicoplanin; - RT
*3*	c	25	Hemoptysis	*Pseudomonas aeruginosa*	2	- Bronchoscopy
*4*	87			*Burkholderia cepacia, Pseudomonas aeruginosa, Staphylococcus aureus*		
*Admission #1*		16	Pneumonia^b^		12	- Clarithromycin
*Admission #2*		31	RE		11	- Oxacillin; - Ceftazidime; - RT
*Admission #3*		38	RE		13	- Oxacillin; - Ceftazidime; - Spontaneous vaginal delivery

Abbreviations: FEV1, predicted forced expiratory volume in 1 second; GA, gestational age; ICU, intensive care unit; MRSA, methicillin-resistant *Staphylococcus aureus*; RE, respiratory exacerbation; RT, respiratory therapy.

a Note that patients also received antithrombotic prophylaxis (enoxaparin), except for the patient with hemoptysis.

b This patient had a medical history of recurrent chest infection and received the diagnosis of cystic fibrosis during the pregnancy.

c Patient had a unilateral pulmonary resection.

When analyzing the lung function parameters, we excluded two pregnancies of the same patient, who underwent a unilateral pulmonary resection. The FEV1 and FVC measures were 38% (±24.3%) and 58% (±15.9%) of the predicted values, respectively. All of the cases with an FEV1 < 50% (*n* = 3) involved multiple RE episodes with a median RE frequency of 4 (2–4) events per patient during the pregnancy.

## Discussion

The prognosis of pregnancy in women with CF has changed, and recent data suggest that CF patients can tolerate pregnancy without poor maternal or fetal outcomes.^16^ Our findings corroborate these data because we have not observed any cases of miscarriage, stillbirth, spontaneous prematurity, neonatal admission to the intensive care unit, small for gestational age newborns, or maternal death in a subset of patients with impaired respiratory function.

Early data regarding pregnant women with CF and severe lung function impairment were dismal. An FEV1 < 50% had once been recommended as an indication for termination of the pregnancy.^17^ Thorpe-Beeston et al^9^ analyzed a retrospective cohort of pregnant women with CF and observed that 3 out of 5 patients with a predicted FEV1 < 40% died within 18 months after the delivery. Gilljam et al^10^ concluded that an FEV1 < 50% was related to a shorter survival. Severe lung impairment has been suggested as being associated with low birthweight and prematurity.^9^ ^14^ ^15^

Our study population did not include any cases of spontaneous prematurity or low birthweight newborns, despite the low FEV1 and a median weight gain of 4.1 kg among the patients. Our findings are consistent with those from a recent French case-control study, in which CF patients and healthy controls had similar mean age of delivery and prematurity rate, as well as newborns with similar birthweight.^14^

Regarding the route of delivery, we have observed a rate of 75% for operative vaginal delivery among the patients who gave birth vaginally. Labor progress, fetal monitoring, and maternal clinical conditions were individually assessed to define the appropriate route of delivery. Our indications for forceps delivery were maternal exhaustion and rotational instrumental delivery. However, it has been previously advised that women with CF and severe lung disease should have the second stage of labor shortened in order to prevent prolonged Valsalva maneuvers.^18^

Burden et al^19^ achieved 100% of vaginal deliveries and 3 cases of operative vaginal deliveries among 15 pregnant women with CF. However, their study population had higher FEV1 values than ours, and 7 out of 11 patients had an FEV1 > 50% at admission. Regarding severe lung disease, Thorpe-Beeston et al^9^ observed that pregnant women with an FEV1 < 60% had a higher likelihood of cesarean delivery.

Pregnant women with CF tended to present with less frequent spontaneous labor and higher rates of operative vaginal delivery than healthy controls in a retrospective case-control study of 33 pregnancies in women with CF.^13^

The most common negative outcomes in our study were hospital admission and RE. Despite our small population, we have observed that 57.1% of the admissions lasted between 2 and 36 days. A retrospective chart review of 43 pregnancies in an American CF referral center found that 100% of the patients required hospitalization for RE or for parenteral nutrition.^14^ A large retrospective review of 216 pregnant women with CF enrolled in the Epidemiologic Study of Cystic Fibrosis showed an increased tendency of hospital admissions during pregnancy, although this was not statistically significant.^20^

Recurrent RE was a ubiquitous event in our study population. Likewise, Gillet et al^21^ described that 77.5% of the pregnant women in the French CF Registry received intravenous antibiotic therapy. Similar to our findings, Cheng et al,^14^ by analyzing 15 pregnant women who had received prenatal care at their CF center, described an inpatient stay of 19 ± 21 days, and a frequency of 2 ± 2 of RE per patient.

Pregnant women with CF required more prenatal visits, had higher hospitalization rates, and underwent lengthier intravenous antibiotic therapy than healthy women.^14^ ^15^ ^20^ Chronic chest infection and recurrent RE are common complications of CF patients with lung disease.^5^ Cystic fibrosis patients tend to be chronically colonized by pathogenic agents such as *P. aeruginosa, Staphylococcus aureus*, and *B. cepacia*.^5^

Pregnancy has been demonstrated to increase the severity of chronic *P. aeruginosa* lung infection in immunomodulated mice.^22^ Airway colonization by *B. cepacia* is known to be related to severe lung impairment and to poor maternal outcomes.^10^ Despite our small series of cases, we have observed that patients colonized by *B. cepacia* had the longest periods of hospitalization.

During the course of pregnancy, acute respiratory infections are particularly critical, as they may reduce lung function and lead to a maternal clinical status resulting in hypoxia.^18^ Animal models have suggested that maternal hypoxia might be related to trophoblast cell invasion, increased risk of maternal hypertension, placental insufficiency, and fetal distress.^23^

Furthermore, pregnancy poses a challenge for lung disease patients. Maternal physiological changes during pregnancy include increases in blood volume and cardiac output, elevation of the diaphragm, decreases in residual volume and functional residual capacities, respiratory alkalosis, hyperventilation, and increased oxygen consumption.^24^ These changes result in a lack of adaptive respiratory capacity and in an increased risk of respiratory failure.^25^

A team of CF respiratory specialists and obstetricians with experience with respiratory diseases and pathological pregnancies should assist pregnant women with CF to guarantee an adequate early diagnosis and the management of RE. Hospital admissions might be more frequent due to the risks of maternal respiratory failure and fetal distress. Courses of intravenous antibiotic therapy will likely be required during pregnancy, and their administration should not be postponed due to the possibility of harmful effects on the fetus.^14^ ^15^ ^18^ ^21^ Sufficient medical data have confirmed the safety of the most common antibiotic agents for lung infection during pregnancy (β-lactam and conventional doses of aminoglycosides).^15^ ^18^

Although previous studies have examined the outcomes of pregnancy in CF patients with mild to moderate lung impairment, our study population consisted mainly of patients with severe lung dysfunction.^9^ ^12^ These findings might result in selection bias, because we have collected data from a referral center.

We have performed a single referral center study; consequently, a weakness of our report is its small number of cases. Importantly, we excluded the FEV1 measures of two pregnancies of the same patient who underwent a unilateral pulmonary resection, which might have hampered our analysis.

## Conclusion

Our findings corroborate the current belief that even women with CF and severe lung impairment can achieve a successful term pregnancy. It is pivotal to note that REs may occur during the course of the pregnancy. Cystic fibrosis patients might require prompt hospital admission to avoid both fetal distress and maternal respiratory compromise. They should also be followed by a specialized team with experience with respiratory diseases. Multicenter prospective studies are still warranted to properly define the odds of obstetric outcomes and maternal risks.

## References

[1] Rosenstein B J Cutting G R; Cystic Fibrosis Foundation Consensus Panel. The diagnosis of cystic fibrosis: a consensus statement J Pediatr 1998 132 04 589 595. Doi: 10.1016/S0022-3476(98)70344-0 9580754 10.1016/s0022-3476(98)70344-0

[2] Raskin S Pereira-Ferrari L Reis F C Incidence of cystic fibrosis in five different states of Brazil as determined by screening of p.F508del, mutation at the CFTR gene in newborns and patients J Cyst Fibros 2008 7 01 15 22. Doi: 10.1016/j.jcf.2007.03.006 17544945 10.1016/j.jcf.2007.03.006

[3] Rowe S M Miller S Sorscher E J Cystic fibrosis N Engl J Med 2005 352 19 1992 2001. Doi: 10.1056/NEJMra043184 15888700 10.1056/NEJMra043184

[4] Ratjen F Döring G Cystic fibrosis Lancet 2003 361(9358):681 689. Doi: 10.1016/S0140-6736(03)12567-6 12606185 10.1016/S0140-6736(03)12567-6

[5] Cystic Fibrosis Foundation. *Patient Registry 2015: Annual Data Report* Bethesda, MD Cystic Fibrosis Foundation 2016

[6] Dodge J A Lewis P A Stanton M Wilsher J Cystic fibrosis mortality and survival in the UK: 1947-2003 Eur Respir J 2007 29 03 522 526. Doi: 10.1183/09031936.00099506 17182652 10.1183/09031936.00099506

[7] Fair A Griffiths K Osman L M. The Collaborative Group of Scottish Adult CF Centres Attitudes to fertility issues among adults with cystic fibrosis in Scotland Thorax 2000 55 08 672 677. Doi: 10.1136/thorax.55.8.672 10899244 10.1136/thorax.55.8.672 PMC1745836

[8] Siegel B Siegel S Pregnancy and delivery in a patient with cystic fibrosis of the pancreas Obstet Gynecol 1960 16 438 440

[9] Thorpe-Beeston J G Madge S Gyi K Hodson M Bilton D The outcome of pregnancies in women with cystic fibrosis–single centre experience 1998-2011 BJOG 2013 120 03 354 361. Doi: 10.1111/1471-0528.12040 23145929 10.1111/1471-0528.12040

[10] Gilljam M Antoniou M Shin J Dupuis A Corey M Tullis D E Pregnancy in cystic fibrosis. Fetal and maternal outcome Chest 2000 118 01 85 91. Doi: 10.1378/chest.118.1.85 10893364 10.1378/chest.118.1.85

[11] Cohen L F di Sant'Agnese P A Friedlander J Cystic fibrosis and pregnancy. A national survey Lancet 1980 2(8199):842 844. Doi: 10.1016/S0140-6736(80)90183-X 6107509 10.1016/s0140-6736(80)90183-x

[12] Goss C H Rubenfeld G D Otto K Aitken M L The effect of pregnancy on survival in women with cystic fibrosis Chest 2003 124 04 1460 1468. Doi: 10.1378/chest.124.4.1460 14555580 10.1378/chest.124.4.1460

[13] Girault A Blanc J Gayet V Goffinet F Hubert D Maternal and perinatal outcomes of pregnancies in women with cystic fibrosis--A single centre case-control study Respir Med 2016 113 22 27. Doi: 10.1016/j.rmed.2016.02.010 27021576 10.1016/j.rmed.2016.02.010

[14] Cheng E Y Goss C H McKone E F Aggressive prenatal care results in successful fetal outcomes in CF women J Cyst Fibros 2006 5 02 85 91. Doi: 10.1016/j.jcf.2006.01.002 16650742 10.1016/j.jcf.2006.01.002

[15] Tonelli M R Aitken M L Pregnancy in cystic fibrosis Curr Opin Pulm Med 2007 13 06 537 540. Doi: 10.1097/MCP.0b013e3282f01120 17901761 10.1097/MCP.0b013e3282f01120

[16] Salvatore D Buzzetti R Baldo E An overview of international literature from cystic fibrosis registries. Part 4: update 2011 J Cyst Fibros 2012 11 06 480 493. Doi: 10.1016/j.jcf.2012.07.005 22884375 10.1016/j.jcf.2012.07.005

[17] Larsen J W Jr Cystic fibrosis and pregnancy Obstet Gynecol 1972 39 06 880 883 5049902

[18] Edenborough F P Borgo G Knoop C Guidelines for the management of pregnancy in women with cystic fibrosis J Cyst Fibros 2008 7 01 S2 S32. Doi: 10.1016/j.jcf.2007.10.001 18024241 10.1016/j.jcf.2007.10.001

[19] Burden C Ion R Chung Y Henry A Downey D G Trinder J Current pregnancy outcomes in women with cystic fibrosis Eur J Obstet Gynecol Reprod Biol 2012 164 02 142 145. Doi: 10.1016/j.ejogrb.2012.06.013 22784584 10.1016/j.ejogrb.2012.06.013

[20] McMullen A H Pasta D J Frederick P D Impact of pregnancy on women with cystic fibrosis Chest 2006 129 03 706 711. Doi: 10.1378/chest.129.3.706 16537871 10.1378/chest.129.3.706

[21] Gillet D de Braekeleer M Bellis G Durieu I; French Cystic Fibrosis Registry. Cystic fibrosis and pregnancy. Report from French data (1980-1999) BJOG 2002 109 08 912 918. Doi: 10.1111/j.1471-0528.2002.01511.x 12197372 10.1111/j.1471-0528.2002.01511.x

[22] Moser C Johannesson M Song Z J Hougen H P Kharazmi A Høiby N Altered cytokine response and increased degree of lung pathology in pregnant C3HIHeN mice with chronic Pseudomonas aeruginosa lung infection Neth J Med 1999 54 S70 S71. Doi: 10.1016/S0300-2977(99)90236-X

[23] Thompson L P Pence L Pinkas G Song H Telugu B P Placental hypoxia during early pregnancy causes maternal hypertension and placental insufficiency in the hypoxic guinea pig model Biol Reprod 2016 95 06 128. Doi: 10.1095/biolreprod.116.142273 27806942 10.1095/biolreprod.116.142273 PMC5315426

[24] Hegewald M J Crapo R O Respiratory physiology in pregnancy Clin Chest Med 2011 32 01 1 13. Doi: 10.1016/j.ccm.2010.11.001 21277444 10.1016/j.ccm.2010.11.001

[25] Mighty H E Acute respiratory failure in pregnancy Clin Obstet Gynecol 2010 53 02 360 368. Doi: 10.1097/GRF.0b013e3181deb3f1 20436311 10.1097/GRF.0b013e3181deb3f1

